# Retraction: Biogenic green synthesis of MgO nanoparticles using *Saussurea costus* biomasses for a comprehensive detection of their antimicrobial, cytotoxicity against MCF-7 breast cancer cells and photocatalysis potentials

**DOI:** 10.1371/journal.pone.0351977

**Published:** 2026-06-22

**Authors:** The PLOS One Editors

After this article [1] was published, concerns were raised regarding results presented in Figs 2-3, 6, and 8-10.

Specifically:

- The following panels appear to fully or partially overlap:
  - Fig 2a in [1], Fig 3a in [2], and Fig 5a in [3]
  - Fig 2b in [1] and Fig 3b in [2]
  - Fig 3A panel a and Fig 3A panel b in [1]
  - Fig 8B a-d in [1] and Fig 2A-D in [4]*
  - Fig 9B Control, MgONPs, and Paclitaxel in [1] and Fig 3A MnP, dox+MnP, and dox in [5]*
  - Fig 9D Control, MgONPs, and Paclitaxel in [1] and Fig 3A Hep2 DG-48h, A431 TQ-24h, and A431 DG-24h in [6]
  - Fig 10A Control, MgONPs, and Paclitaxel in [1] and Fig 7A Con, DZ, and DZ + MgO NPs in [7]*
  - Fig 10D MgONPs 24h and Fig 10D Control 24h in [1]
  - Fig 10D MgONPs 72h and Fig 10D Control 48h in [1]
- There appear to be repetitive areas within one or more panels in the following figures in [1]:
  - Fig 6
  - Fig 10D
- Fig 9A in [1] appears similar but not identical to Fig 11D in [8], and there are areas of the background in Fig 9A in [1] that appear discontinuous with the surrounding areas.

Regarding Fig 3A panels a and b, the corresponding author stated that the visual similarity between the two profiles, specifically the positions of the characteristic peaks for magnesium and oxygen, is expected as both panels characterize magnesium oxide nanoparticles synthesized using the same underlying chemical structure. The corresponding author stated they did not agree with the above concerns for Fig 6, Fig 9A, and Fig 10D Control 72h panel.

Regarding the above similarities between Figs 2a and 2b in [1], Figs 3a and 3b in [2], and Fig 5a in [3], the corresponding author stated that Figs 2a and 2b in [1] are correct and that Figs 3a and 3b in [2] and Fig 5a in [3] are incorrect.

The corresponding author provided incomplete underlying image data for Figs 2-3, 6, and 8-10. Upon editorial review, the comments and data provided by the authors in response to the above-mentioned issues did not resolve the concerns about the published results.

In light of the above concerns that question the reliability and integrity of the reported results, the *PLOS One* Editors retract this article.

MA did not agree with the retraction. NMAM, NAA, MFET, HFO, GAAH, SIB, and NMSM either did not respond directly or could not be reached.

The Fig 8B panels a-d, Fig 9 A, B, and D, and Fig 10A Control, MgONPs, and Paclitaxel appear similar to previously published materials by different authors that were not offered under a CC BY license, and are therefore excluded from this article’s [1] license. The * by the citations above marks the oldest publication of each respective image of which PLOS is aware.

## References

[1] Amina M, Al Musayeib NM, Alarfaj NA, El-Tohamy MF, Oraby HF, Al Hamoud GA, et al. RETRACTED: Biogenic green synthesis of MgO nanoparticles using *Saussurea costus* biomasses for a comprehensive detection of their antimicrobial, cytotoxicity against MCF-7 breast cancer cells and photocatalysis potentials. PLoS One. 2020;15(8):e0237567. doi: 10.1371/journal.pone.0237567 32797097 PMC7428194

[2] Amina M, Al Musayeib NM, Alarfaj NA, El-Tohamy MF, Al-Hamoud GA, Al-Yousef HM. Immunomodulatory and Antioxidant Potential of Biogenic Functionalized Polymeric Nutmeg Oil/Polyurethane/ZnO Bionanocomposite. Pharmaceutics. 2021;13(12):2197. doi: 10.3390/pharmaceutics13122197 34959478 PMC8703756

[3] Alarfaj NA, Alabdulmonem HA, Al-Onazi WA, Al-Mohaimeed AM, El-Tohamy MF. Biogenic synthesis of ZnO and Al2O3 nanoparticles using *Camellia sinensis* and *Origanum vulgare* L. leaves extract for spectroscopic estimation of ofloxacin and ciprofloxacin in commercial formulations. PLoS One. 2023;18(10):e0286341. doi: 10.1371/journal.pone.0286341 37906583 PMC10617719

[4] Noureini SK, Esmaili H. Multiple mechanisms of cell death induced by chelidonine in MCF-7 breast cancer cell line. Chem Biol Interact. 2014;223:141–9. doi: 10.1016/j.cbi.2014.09.013 25265580

[5] Flórido A, Saraiva N, Cerqueira S, Almeida N, Parsons M, Batinic-Haberle I, et al. The manganese(III) porphyrin MnTnHex-2-PyP5+ modulates intracellular ROS and breast cancer cell migration: Impact on doxorubicin-treated cells. Redox Biol. 2019;20:367–78. doi: 10.1016/j.redox.2018.10.016 30408752 PMC6222139

[6] Das S, Dey KK, Dey G, Pal I, Majumder A, MaitiChoudhury S, et al. Antineoplastic and apoptotic potential of traditional medicines thymoquinone and diosgenin in squamous cell carcinoma. PLoS One. 2012;7(10):e46641. doi: 10.1371/journal.pone.0046641 23077516 PMC3471895

[7] Shiri M, Navaei-Nigjeh M, Baeeri M, Rahimifard M, Mahboudi H, Shahverdi AR, et al. Blockage of both the extrinsic and intrinsic pathways of diazinon-induced apoptosis in PaTu cells by magnesium oxide and selenium nanoparticles. Int J Nanomedicine. 2016;11:6239–50. doi: 10.2147/IJN.S119680 27920530 PMC5125760

[8] Amina M, Al Musayeib NM, Al-Hamoud GA, Al-Dbass A, El-Ansary A, Ali MA. Prospective of biosynthesized L.satiVum oil/PEG/Ag-MgO bionanocomposite film for its antibacterial and anticancer potential. Saudi Journal of Biological Sciences. 2021;28:5971–85. doi: 10.1016/j.sjbs.2021.06.052 34588914 10.1016/j.sjbs.2021.06.052 PMC8459159

